# Diabesity phenotype in relation to the incidence and resolution of nonalcoholic fatty liver disease: A prospective cohort study

**DOI:** 10.1111/1753-0407.13459

**Published:** 2023-08-16

**Authors:** Lijie Kong, Chaojie Ye, Yiying Wang, Chun Dou, Jie Zheng, Shuangyuan Wang, Hong Lin, Zhiyun Zhao, Mian Li, Yu Xu, Yuhong Chen, Jieli Lu, Min Xu, Weiqing Wang, Guang Ning, Yufang Bi, Tiange Wang

**Affiliations:** ^1^ Department of Endocrine and Metabolic Diseases, Shanghai Institute of Endocrine and Metabolic Diseases, Ruijin Hospital Shanghai Jiao Tong University School of Medicine Shanghai China; ^2^ Shanghai National Clinical Research Center for Metabolic Diseases, Key Laboratory for Endocrine and Metabolic Diseases of the National Health Commission of the PR China, Shanghai Key Laboratory for Endocrine Tumor, Ruijin Hospital Shanghai Jiao Tong University School of Medicine Shanghai China

**Keywords:** diabesity, nonalcoholic fatty liver disease, obesity, prediabetes, type 2 diabetes

## Abstract

**Background:**

Diabesity is a term used to emphasize the dual epidemic and the combined detrimental effects of diabetes and obesity. We aimed to investigate the associations of diabesity with the incidence and resolution of nonalcoholic fatty liver disease (NAFLD).

**Methods:**

This prospective cohort study included 5549 participants with a median follow‐up of 4.3 years (2010–2015). Diabesity was defined as six categories by the combinations of glucose tolerance status (normal glucose tolerance [NGT], prediabetes, and diabetes) diagnosed by fasting and oral glucose tolerance test 2‐h glucose and hemoglobin A1c and general or abdominal obesity status. We examined the odds ratios (ORs) for the incidence and resolution of NAFLD associated with diabesity categories, respectively.

**Results:**

For NAFLD incidence, compared with the diabesity category of NGT with nonobesity, the categories of either glucose intolerance or general obesity were associated with higher risks of NAFLD, of which the categories with obesity, regardless of glucose intolerance status, exhibited greater risks (ORs ranged from 3.19 to 4.49) than the categories of nonobesity. For NAFLD resolution, the categories of prediabetes or diabetes with obesity were associated with decreased likelihoods of a resolution of NAFLD (ORs ranged from 0.40 to 0.58). These association patterns were consistent across various definitions of diabesity by glucose tolerance status diagnosed by different combinations of glycemic parameters and general or abdominal obesity.

**Conclusions:**

The diabesity association pattern with NAFLD incidence was mainly determined by obesity, while that with NAFLD resolution was driven by the combined phenotype of glucose intolerance and obesity.

## Graphical abstract

**Highlights**

- Obesity determines the association pattern of diabesity with the incidence of NAFLD.
- The combined phenotype of glucose intolerance and obesity drives diabesity‐related likelihoods of NAFLD resolution.
- Our findings suggest that diabesity should be incorporated into NAFLD management programs to prioritize risk stratifications and guide personalized strategies for the prevention and control of NAFLD.

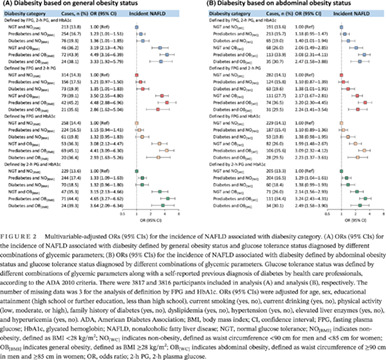

## 1 INTRODUCTION

Nonalcoholic fatty liver disease (NAFLD) is the principal etiology for advanced liver diseases such as cirrhosis and hepatocellular carcinoma.^1^ Globally, NAFLD affects more than 25% of the population,^2^ and its prevalence is rising in parallel with the twin epidemics of type 2 diabetes and obesity, two key risk factors for NAFLD.^3^

Diabesity is a novel phenotype proposed to highlight the dual epidemics and combined health effects of type 2 diabetes and obesity,^4^ because their coexistence and close interplay may synergistically regulate the development of cardiometabolic diseases.^5^, ^6^, ^7^ Elucidating the association patterns between diabesity and NAFLD could help improve the optimal implementation of effective intervention strategies for the prevention and control of NAFLD. Thus far, several uncertainties on this topic are worthy of attention. First, in previous studies, diabesity was generally defined as the combination of type 2 diabetes and obesity.^4^, ^8^ Given the high prevalence of prediabetes and abdominal obesity in China,^9^ it is essential to adopt a comprehensive definition of diabesity that encompasses different glycemic tolerance statuses, including normal glucose tolerance (NGT), prediabetes, and diabetes, as well as different obesity indicators such as body mass index (BMI) and waist circumference, to enable a clear appraisal of the NAFLD risk in individuals with various diabesity phenotypes. Second, NAFLD is partially reversible and preventable. However, it is unclear whether diabesity is associated with both the incidence and resolution of NAFLD and whether diabesity exhibits different association patterns with these NAFLD outcomes.

To this end, we investigated the associations of diabesity phenotypes with the incidence and resolution of NAFLD in a prospective cohort study. Specifically, we defined diabesity phenotypes based on joint categories of glucose tolerance status diagnosed by different combinations of fasting plasma glucose, oral glucose tolerance test (OGTT) 2‐h plasma glucose, and glycated hemoglobin A1c (HbA1c) and obesity status including general and abdominal obesity, to reflect a comprehensive profile of diabesity.

## 2 METHODS

### 2.1 Study design and participants

**FIGURE 1:**
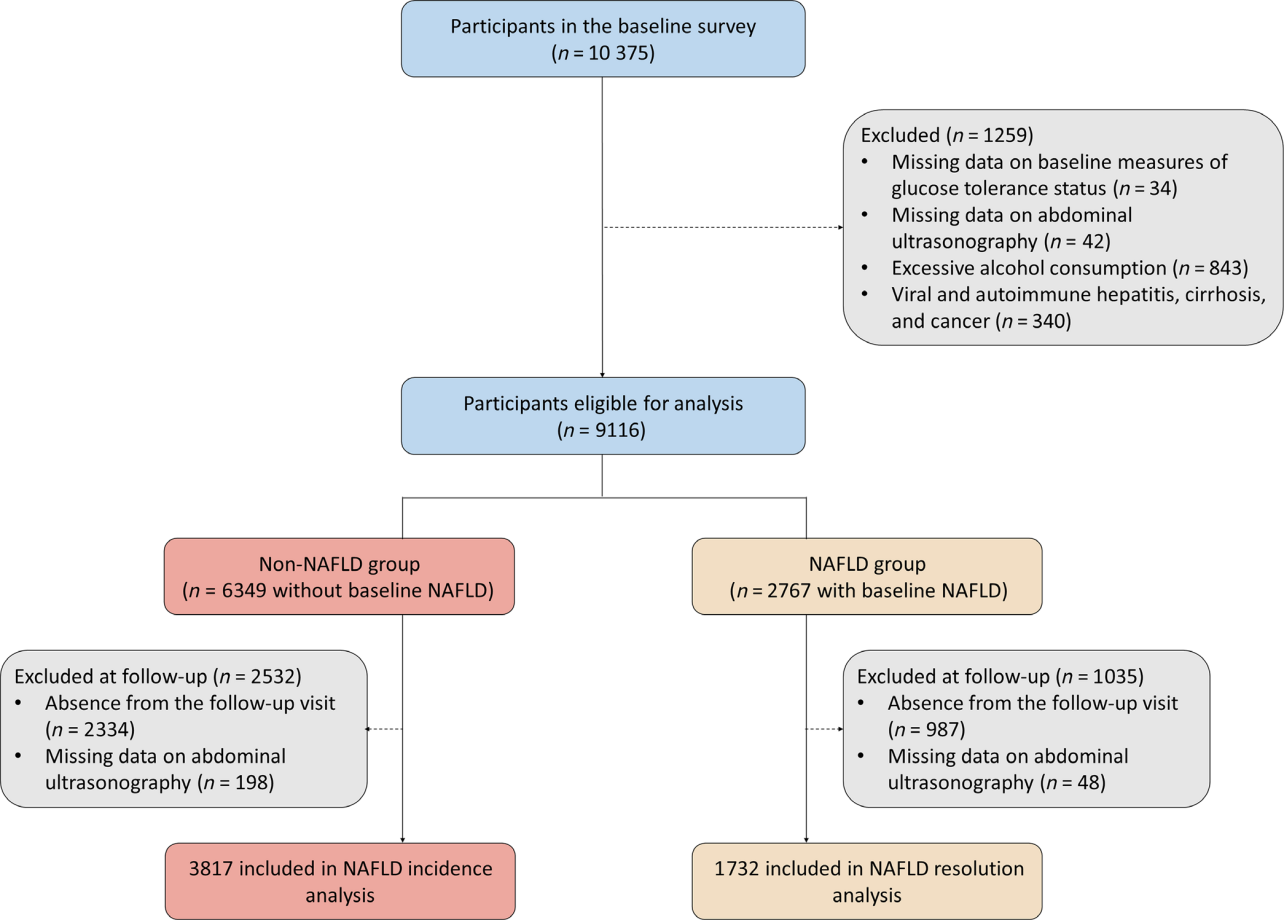
Flow chart of the study participants. NAFLD, nonalcoholic fatty liver disease.

Study participants were recruited from an ongoing population‐based, prospective cohort study in Jiading District, Shanghai, China, as described previously.^10^ Between March and August 2010, a total of 10 375 residents aged ≥40 years from local resident registration systems were recruited and underwent a comprehensive baseline survey comprising a standard questionnaire and clinical measurement of blood and urine samples.^10^ Between August 2014 and July 2015, all participants were invited to attend an in‐person follow‐up survey, which followed the same protocols as the baseline survey. In this study, we excluded 34 participants with missing data on baseline measures of glucose tolerance status, 42 participants with missing data on baseline abdominal ultrasonography, 843 participants with excessive alcohol consumption (alcohol consumption ≥210 g/week in men and ≥140 g/week in women),^11^ and 340 participants with known liver diseases including viral and autoimmune hepatitis, cirrhosis, and cancer. The remaining 9116 participants were divided into two groups according to baseline ultrasound‐detected NAFLD status. We further excluded 3321 participants who were inaccessible to an onsite follow‐up visit and 246 participants with missing data on abdominal ultrasonography at follow‐up. Finally, a total of 5549 participants were included in this study, with 3817 and 1732 participants for the analyses of associations of diabesity phenotype with the incidence and resolution of NAFLD, respectively (Figure 1). This study was approved by the Medical Ethics Committee of Ruijin Hospital, Shanghai Jiao Tong University, Shanghai, China. All study participants provided written informed consent.

### 2.2 Data collection and definition

Baseline and follow‐up surveys were performed in local community clinics by trained study personnel with standardized protocols. Information on demographic characteristics, educational attainment, family history of diabetes, lifestyle behaviors including alcohol consumption, smoking status, physical activity, and medical history were collected by standardized questionnaires. Educational attainment was classified as less than high school (<9 years) and high school or further (≥9 years). Current smokers or drinkers were defined as participants who had consumed cigarettes or alcohol regularly in the past 6 months. The International Physical Activity Questionnaire was used to obtain information about the intensity, duration, and frequency of physical activity, and the metabolic equivalent task (MET) was calculated to evaluate average weekly energy expenditure. Physical activity was classified as low (0–<600 MET‐min/week), moderate (600–<3000 MET‐min/week), and high (≥3000 MET‐min/week) categories.^12^ Family history of diabetes was defined as having at least one first‐degree relative with diabetes. Self‐reported disease and medication were verified by the carry‐on medical records.

Anthropometric measurements, including body weight, height, waist circumference, and blood pressure were measured by trained staff in accordance with standard protocols. BMI was calculated as weight in kilograms divided by the square of the height in meters (kg/m^2^). Waist circumference was measured with a measuring tape placed midway between the lowest rib and the upper border of the iliac crest when the participants exhaled normally in a standing position. Blood pressures were measured three times by an automated electronic device (Omron model HEM‐752 FUZZY; Omron Company, Dalian, China) on the nondominant arm after resting for 5 min in a sitting position, and three measures were averaged for analyses. Hypertension was defined as systolic blood pressure ≥140 mm Hg, diastolic blood pressure ≥90 mm Hg, or taking antihypertensive medications.

All participants fasted for ≥10 h before experiencing a 75‐g OGTT, and blood samples were collected at 0 and 2 h during the test. Fasting and OGTT 2‐h plasma glucose concentrations were measured by the glucose oxidase method using an auto‐analyzer (Modular P800; Roche, Basel, Switzerland) within 2 h after blood sample collection. HbA1c was measured by high‐performance liquid chromatography using the VARIANT II Hemoglobin Testing System (Bio‐Rad Laboratories, Hercules, CA, USA) at the central laboratory at the Shanghai Institute of Endocrine and Metabolic Diseases, which was certificated by the National Glycohemoglobin Standardization Program and the College of American Pathologists Laboratory Accreditation Program. Fasting insulin, total cholesterol, triglycerides, low‐density lipoprotein cholesterol (LDL‐C), high‐density lipoprotein cholesterol (HDL‐C), liver enzymes (including alanine aminotransferase [ALT], aspartate aminotransferase [AST], and γ‐glutamyl‐transferase [GGT]), and serum uric acid were measured on auto‐analyzers (Modular E170; Roche, Basel, Switzerland). The index of homeostasis model assessment of insulin resistance (HOMA‐IR) was calculated as fasting insulin (μIU/mL) × fasting glucose (mmol/L)/22.5.^13^ Dyslipidemia was defined based on the National Cholesterol Education Program guidelines as total cholesterol ≥6.22 mmol/L, LDL‐C ≥ 4.14 mmol/L, HDL‐C < 1.04 mmol/L, triglycerides ≥2.26 mmol/L, or taking lipid‐lowering medications.^14^ Elevated liver enzyme was defined as ALT >50 IU/L, AST >50 IU/L, or GGT >60 IU/L, based on the reference range provided in the laboratory test report. Hyperuricemia was defined as concentrations of serum uric acid ≥360 μmol/L in women and ≥420 μmol/L in men.^15^

### 2.3 Definition of diabesity phenotype

Glucose tolerance status including NGT, prediabetes, and diabetes was defined according to the American Diabetes Association (ADA) 2010 criteria.^16^ Diabetes was defined as fasting plasma glucose ≥126 mg/dL (7.0 mmol/L), or 2‐h plasma glucose ≥200 mg/dL (11.1 mmol/L), or HbA1c ≥6.5%, or a self‐reported previous diagnosis of diabetes by health care professionals. Among participants without diabetes, NGT was defined as fasting plasma glucose <100 mg/dL (5.6 mmol/L), 2‐h plasma glucose <140 mg/dL (7.8 mmol/L), and HbA1c <5.7%; prediabetes was defined as fasting plasma glucose of 100 mg/dL (5.6 mmol/L) to <126 mg/dL (7.0 mmol/L), or 2‐h plasma glucose of 140 mg/dL (7.8 mmol/L) to <200 mg/dL (11.1 mmol/L), or HbA1c of 5.7% to <6.5%. Glucose tolerance status was defined by all three glycemic parameters in the main analyses and by different combinations of each two of the glycemic parameters in the sensitivity analyses.

General obesity was defined as BMI ≥28 kg/m^2^, according to the Working Group on Obesity in China.^17^ Abdominal obesity was defined as waist circumference of ≥90 cm in men and ≥85 cm in women, according to the Chinese Diabetes Society.^18^

Based on glucose tolerance status and general or abdominal obesity status, diabesity was classified into six categories: (1) NGT with nonobesity; (2) NGT with obesity; (3) prediabetes with nonobesity; (4) prediabetes with obesity; (5) diabetes with nonobesity; and (6) diabetes with obesity.

### 2.4 Ascertainment of NAFLD

At baseline and follow‐up surveys, hepatic ultrasonography was performed independently by two clinical sonographers with blindness to clinical and laboratory data, using a high‐resolution B‐mode tomographic ultrasound system (Esaote Biomedica SpA, Genoa, Italy) with a 3.5‐MHz probe. Fatty liver was diagnosed by the presence of at least two of the following abnormal imaging findings: (1) diffusely increased echogenicity of the liver relative to the kidney or spleen, (2) ultrasound beam attenuation, or (3) poor visualization of intrahepatic structures. When the former two diagnoses of fatty liver were inconsistent, a blinded third sonographer was required. NAFLD was defined as ultrasound‐detected fatty liver without excessive alcohol consumption or other hepatic diseases.^19^ Incidence of NAFLD referred to non‐NAFLD at baseline progressed to NAFLD at follow‐up, and resolution of NAFLD referred to NAFLD at baseline regressed to non‐NAFLD at follow‐up.^20^

### 2.5 Statistical analysis

Baseline characteristics of participants according to diabesity categories stratified by baseline NAFLD status were presented as means with SDs or medians with interquartile ranges for continuous variables and numbers with proportions for categorical variables. We used multivariable logistic regression models to estimate odds ratios (ORs) and 95% confidence intervals (CIs) for the incidence and resolution of NAFLD associated with diabesity categories. Multivariable‐adjusted ORs (95% CIs) for the incidence and resolution of NAFLD associated with glucose intolerance (ie, prediabetes and diabetes) and obesity (ie, general and abdominal obesity) were also evaluated. Models were adjusted for age, sex, educational attainment, current smoking, current drinking, physical activity, family history of diabetes, dyslipidemia, hypertension, elevated liver enzymes, and hyperuricemia.

We performed two sensitivity analyses. First, in addition to the glucose tolerance status defined by all three glycemic parameters, we replicated the main analyses using glucose tolerance status defined by different combinations of each two of fasting plasma glucose, OGTT 2‐h plasma glucose, and HbA1c to evaluate the stability of diabesity association patterns with incidence and resolution of NAFLD. Second, taking into account the age‐related mass loss, we repeated the main analyses between diabesity and NAFLD outcomes among participants aged <75 years.

Statistical analyses were performed using SAS version 9.4 (SAS Institute, Cary, NC, USA). A two‐sided *p* value <.05 was considered statistically significant.

## 3 RESULTS

### 3.1 Baseline characteristics

When defining diabesity by three glycemic parameters‐diagnosed glucose tolerance status and general obesity, compared with participants with NGT and nonobesity, participants with glucose intolerance or general obesity were older and were more likely to have lower educational attainment, lower proportions of moderate or high physical activity, and a higher prevalence of medical history (Table 1). Participants with glucose intolerance or general obesity also had worse metabolic profiles, including higher levels of BMI, waist circumference, fasting and OGTT 2‐h plasma glucose, HbA1c, and HOMA‐IR than participants with NGT and nonobesity. Similarly, when defining diabesity by abdominal obesity, compared with participants with NGT and nonobesity, participants with glucose intolerance or abdominal obesity had relatively poor metabolic profiles (Supplementary Table S1).

**TABLE 1:** Baseline characteristics of participants according to diabesity category† stratified by baseline NAFLD status.

Characteristic	Normal glucose tolerance		Prediabetes		Diabetes	
	Nonobesity	General obesity	Nonobesity	General obesity	Nonobesity	General obesity
Non‐NAFLD (*n* = 3817)‡						
Participants, *n* (%)	1546 (40.5)	127 (3.3)	1523 (39.9)	164 (4.3)	394 (10.3)	63 (1.7)
Age, years	54.7 (8.6)	56.3 (8.5)	58.3 (8.7)	58.4 (8.4)	60.6 (8.7)	63.4 (8.4)
Men, *n* (%)	445 (28.8)	44 (34.7)	430 (28.2)	43 (26.2)	153 (38.8)	26 (41.3)
High school or further education, *n* (%)	369 (23.9)	20 (15.8)	270 (17.7)	18 (11.0)	89 (22.6)	9 (14.3)
Current cigarette smoker, *n* (%)	250 (16.2)	14 (11.0)	215 (14.1)	18 (11.0)	61 (15.5)	6 (9.5)
Current alcohol drinker, *n* (%)	38 (2.5)	4 (3.2)	49 (3.2)	4 (2.4)	13 (3.3)	2 (3.2)
Physical activity, *n* (%)						
Low	974 (63.0)	82 (64.6)	1008 (66.2)	119 (72.6)	271 (68.8)	45 (71.4)
Moderate	327 (21.2)	29 (22.8)	331 (21.7)	31 (18.9)	84 (21.3)	13 (20.6)
High	245 (15.9)	16 (12.6)	184 (12.1)	14 (8.5)	39 (9.9)	5 (7.9)
Family history of diabetes, *n* (%)	125 (8.1)	5 (3.9)	123 (8.1)	15 (9.2)	96 (24.4)	9 (14.3)
Dyslipidemia, *n* (%)	437 (28.3)	55 (43.3)	531 (34.9)	65 (39.6)	162 (41.1)	34 (54.0)
Hypertension, *n* (%)	641 (41.5)	94 (74.0)	854 (56.3)	122 (74.4)	268 (68.0)	57 (90.5)
Elevated liver enzymes, *n* (%)	58 (3.8)	13 (10.2)	80 (5.3)	12 (7.3)	33 (8.4)	11 (17.5)
Hyperuricemia, *n* (%)	114 (7.4)	15 (11.8)	140 (9.2)	32 (19.5)	39 (9.9)	12 (19.1)
Medical history, *n* (%)						
Antihypertensive medications	236 (15.3)	46 (36.2)	347 (22.8)	68 (41.5)	134 (34.0)	30 (47.6)
Glucose‐lowering medications	0 (0)	0 (0)	0 (0)	0 (0)	173 (43.9)	35 (55.6)
BMI, kg/m^2^	23.5 (2.3)	29.4 (1.4)	23.8 (2.3)	29.5 (1.3)	23.9 (2.3)	29.6 (1.9)
Waist circumference, cm	77.6 (7.0)	89.8 (5.5)	78.7 (6.9)	90.5 (5.6)	80.7 (6.5)	91.5 (6.4)
Fasting plasma glucose, mmol/L	4.8 (0.4)	4.8 (0.4)	5.3 (0.6)	5.3 (0.6)	7.2 (2.3)	7.2 (1.9)
OGTT 2‐h plasma glucose, mmol/L	5.7 (1.1)	5.8 (1.3)	7.1 (1.8)	7.3 (1.7)	14.5 (5.5)	14.6 (4.7)
HbA1c, %	5.3 (0.2)	5.4 (0.2)	5.7 (0.3)	5.8 (0.2)	6.8 (1.5)	6.8 (1.3)
HOMA‐IR	1.2 (0.8–1.6)	1.7 (1.2–2.2)	1.4 (1.0–1.9)	1.9 (1.2–2.6)	1.9 (1.2–2.9)	2.8 (2.0–4.2)
NAFLD (*n* = 1732)§						
Participants, *n* (%)	198 (11.4)	132 (7.6)	465 (26.9)	314 (18.1)	366 (21.1)	257 (14.8)
Age, years	54.2 (7.3)	55.5 (8.1)	57.5 (7.9)	57.5 (8.2)	59.1 (8.5)	58.9 (8.2)
Men, *n* (%)	72 (36.4)	43 (32.6)	136 (29.3)	77 (24.5)	134 (36.6)	77 (30.0)
High school or further education, *n* (%)	59 (29.8)	28 (21.2)	86 (18.5)	59 (18.8)	81 (22.1)	34 (13.2)
Current cigarette smoker, *n* (%)	36 (18.2)	21 (15.9)	70 (15.1)	35 (11.2)	64 (17.5)	46 (17.9)
Current alcohol drinker, *n* (%)	9 (4.6)	3 (2.3)	17 (3.7)	8 (2.6)	11 (3.0)	14 (5.5)
Physical activity, *n* (%)						
Low	126 (63.6)	81 (61.4)	315 (67.7)	218 (69.4)	253 (69.1)	184 (71.6)
Moderate	45 (22.7)	27 (20.5)	94 (20.2)	56 (17.8)	70 (19.1)	51 (19.8)
High	27 (13.6)	24 (18.2)	56 (12.0)	40 (12.7)	43 (11.8)	22 (8.6)
Family history of diabetes, *n* (%)	25 (12.6)	13 (9.9)	59 (12.7)	32 (10.2)	76 (20.8)	61 (23.7)
Dyslipidemia, *n* (%)	120 (60.6)	73 (55.3)	288 (61.9)	181 (57.6)	235 (64.2)	166 (64.6)
Hypertension, *n* (%)	105 (53.0)	98 (74.2)	324 (69.7)	254 (80.9)	279 (76.2)	221 (86.3)
Elevated liver enzymes, *n* (%)	21 (10.6)	19 (14.4)	64 (13.8)	54 (17.2)	65 (17.8)	91 (35.4)
Hyperuricemia	44 (22.2)	30 (22.7)	90 (19.4)	89 (28.3)	84 (23.0)	74 (28.8)
Medical history, *n* (%)						
Antihypertensive medications	38 (19.2)	56 (42.4)	163 (35.1)	147 (46.8)	146 (39.9)	138 (53.7)
Glucose‐lowering medications	0 (0)	0 (0)	0 (0)	0 (0)	130 (35.5)	74 (28.8)
BMI, kg/m^2^	25.5 (1.6)	30 (1.9)	25.6 (1.6)	30.3 (2.0)	25.6 (1.7)	30.8 (2.6)
Waist circumference, cm	85 (5.4)	93 (6.4)	84.3 (5.9)	93.4 (6.3)	85.7 (5.8)	95.9 (7.8)
Fasting plasma glucose, mmol/L	4.9 (0.4)	5 (0.4)	5.4 (0.6)	5.4 (0.6)	7.7 (2.7)	7.6 (2.5)
OGTT 2‐h plasma glucose, mmol/L	6.1 (1.1)	6.3 (1.1)	7.9 (1.7)	7.9 (1.6)	15.8 (5.4)	15 (4.8)
HbA1c, %	5.3 (0.2)	5.4 (0.2)	5.8 (0.3)	5.8 (0.3)	7.2 (1.6)	7.1 (1.4)
HOMA‐IR	1.8 (1.3–2.4)	2.4 (1.7–3.1)	2.2 (1.6–3.0)	2.8 (2.1–3.8)	3.1 (2.1–4.7)	4.2 (2.9–6.4)

*Note*: Data are mean (SD) for continuous variables with normal distribution, median (interquartile range) for continuous variables with skewed distribution, and number (proportion) for categorical variables.

Abbreviations: ADA, American Diabetes Association; BMI, body mass index; HbA1c, glycated hemoglobin; HOMA‐IR, homeostasis model assessment of insulin resistance; NAFLD, nonalcoholic fatty liver disease; OGTT, oral glucose tolerance test.

^†^ Diabesity categories were based on three glycemic parameters‐defined glucose tolerance status according to the ADA criteria 2010 and abdominal obesity.

^‡^ The number of missing data was 1 for waist circumference, 3 for 2‐h plasma glucose, and 4 for HbA1c.

^§^ The number of missing data was 1 for waist circumference, 2 for 2‐h plasma glucose, and 2 for HbA1c.

### 3.2 Association between diabesity and the incidence of NAFLD

**FIGURE 2:**
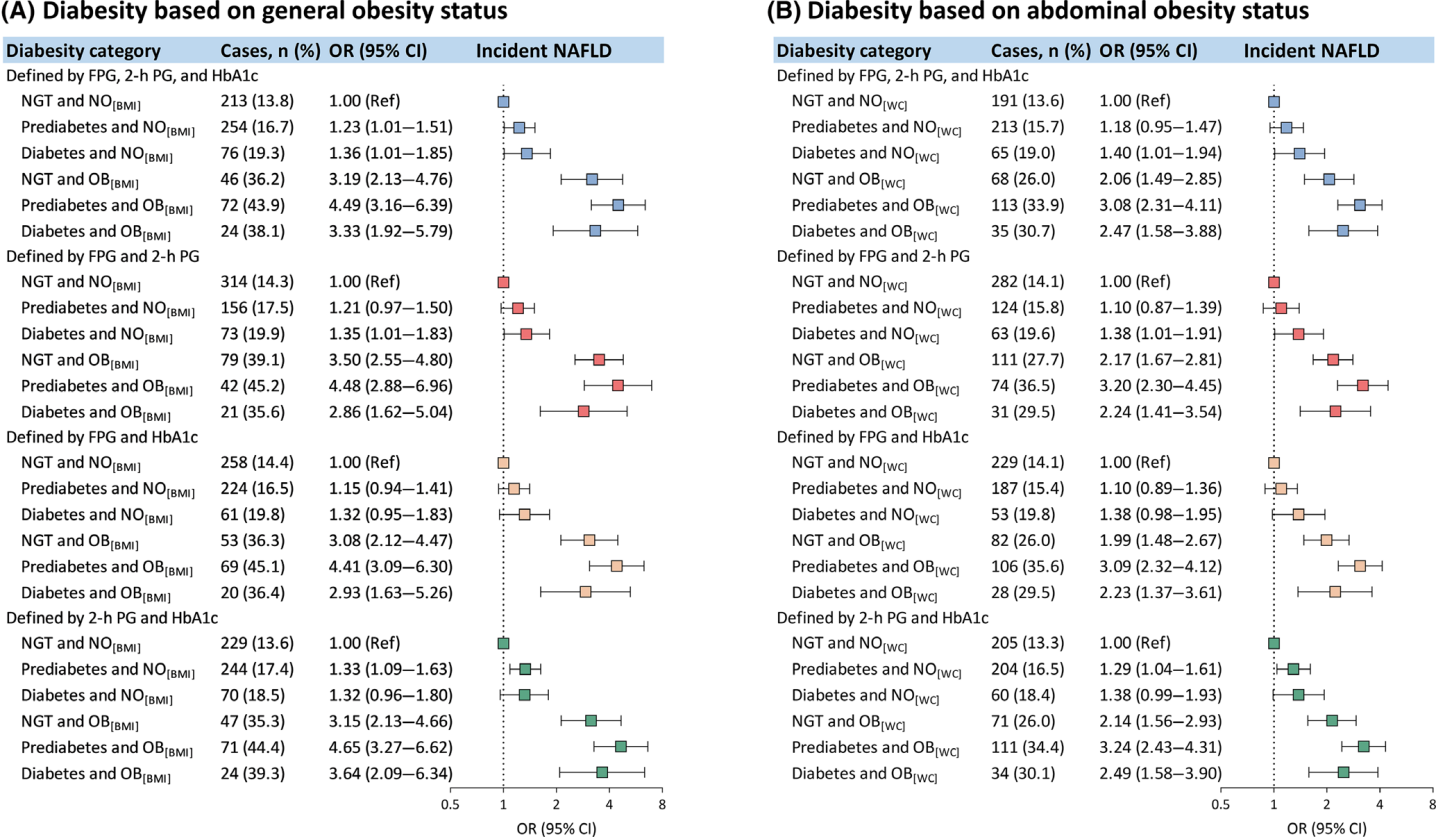
Multivariable‐adjusted ORs (95% CIs) for the incidence of NAFLD associated with diabesity category. (A) ORs (95% CIs) for the incidence of NAFLD associated with diabesity defined by general obesity status and glucose tolerance status diagnosed by different combinations of glycemic parameters; (B) ORs (95% CIs) for the incidence of NAFLD associated with diabesity defined by abdominal obesity status and glucose tolerance status diagnosed by different combinations of glycemic parameters. Glucose tolerance status was defined by different combinations of glycemic parameters along with a self‐reported previous diagnosis of diabetes by health care professionals, according to the ADA 2010 criteria. There were 3817 and 3816 participants included in analysis (A) and analysis (B), respectively. The number of missing data was 3 for the analysis of definition by FPG and HbA1c. ORs (95% CIs) were adjusted for age, sex, educational attainment (high school or further education, less than high school), current smoking (yes, no), current drinking (yes, no), physical activity (low, moderate, or high), family history of diabetes (yes, no), dyslipidemia (yes, no), hypertension (yes, no), elevated liver enzymes (yes, no), and hyperuricemia (yes, no). ADA, American Diabetes Association; BMI, body mass index; CI, confidence interval; FPG, fasting plasma glucose; HbA1c, glycated hemoglobin; NAFLD, nonalcoholic fatty liver disease; NGT, normal glucose tolerance; NO_[BMI]_ indicates non‐obesity, defined as BMI <28 kg/m^2^; NO_[WC]_ indicates non‐obesity, defined as waist circumference <90 cm for men and <85 cm for women; OB_[BMI]_ indicates general obesity, defined as BMI ≥28 kg/m^2^; OB_[WC]_ indicates abdominal obesity, defined as waist circumference of ≥90 cm in men and ≥85 cm in women; OR, odds ratio; 2‐h PG, 2‐h plasma glucose.

During a median of 4.3 years follow‐up, 685 (17.9%) participants developed NAFLD. Prediabetes (multivariable‐adjusted OR 1.26; 95% CI 1.05–1.52), diabetes (1.35; 1.03–1.78), general obesity (3.28; 2.58–4.17), and abdominal obesity (2.27; 1.87–2.76) were independently associated with an increased risk of NAFLD (Supplementary Table S2). As for diabesity defined by three glycemic parameters‐diagnosed glucose tolerance status and general obesity, compared with the diabesity category of NGT with nonobesity, all other five categories (with either glucose intolerance or general obesity) were associated with higher risks of NAFLD (Figure 2A), of which the three categories with obesity (ORs ranged from 3.19 to 4.49) exhibited greater risks than the categories with glucose intolerance and nonobesity (ORs ranged from 1.23 to 1.36). Notably, even the category of NGT with obesity showed a higher risk of NAFLD (OR 3.19; 95% CI 2.13–4.76) than the category of diabetes with nonobesity (1.36; 1.01–1.85). These association patterns were similar across various definitions of glucose tolerance status diagnosed by different combinations of each two of the glycemic parameters.

When three glycemic parameters‐diagnosed glucose tolerance status and abdominal obesity were used to define diabesity, the categories of diabetes with nonobesity (OR 1.40; 95% CI 1.01–1.94) and the categories with abdominal obesity regardless of glucose tolerance status (ORs ranged from 2.06 to 3.08) were associated with increased risks for NAFLD, compared with the category of NGT with nonobesity (Figure 2B). Across various definitions of glucose tolerance status diagnosed by different combinations of glycemic parameters, the category of prediabetes with abdominal obesity conferred the highest risk of NAFLD in magnitude, whereas the category of prediabetes with nonobesity showed no excess risk, except for glucose tolerance status defined by 2‐h plasma glucose and HbA1c.

To control for the possible influence of age‐related mass loss, we replicated the main analyses in participants aged <75 years and observed similar association patterns of diabesity with incident NAFLD (Supplementary Tables S3 and S4).

### 3.3 Association between diabesity and the resolution of NAFLD

**FIGURE 3:**
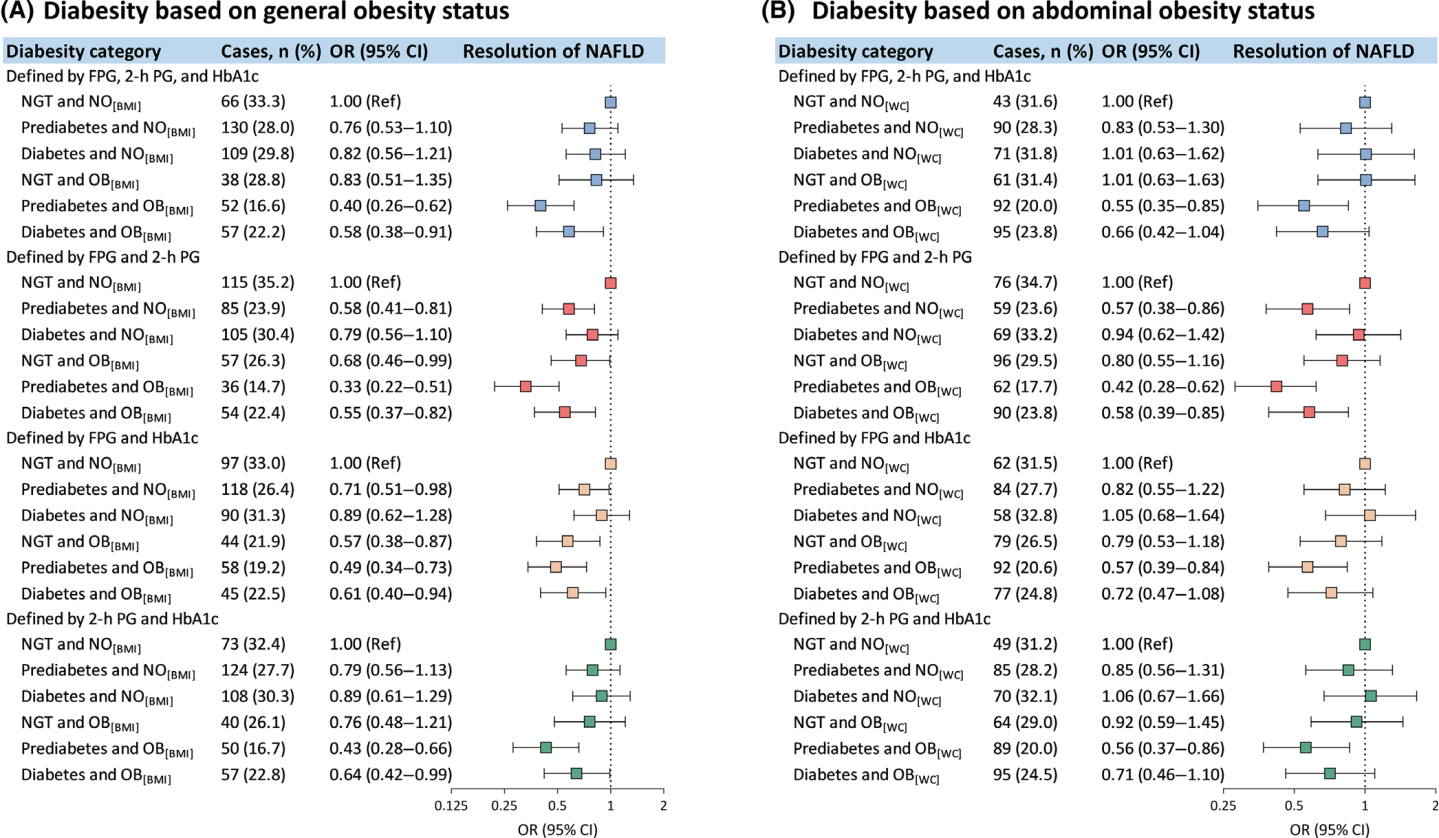
Multivariable‐adjusted ORs (95% CIs) for the resolution of NAFLD associated with diabesity category. (A) ORs (95% CIs) for the resolution of NAFLD associated with diabesity defined by general obesity status and glucose tolerance status diagnosed by different combinations of glycemic parameters; (B) ORs (95% CIs) for the resolution of NAFLD associated with diabesity defined by abdominal obesity status and glucose tolerance status diagnosed by different combinations of glycemic parameters. Glucose tolerance status was defined by different combinations of glycemic parameters along with a self‐reported previous diagnosis of diabetes by health care professionals, according to the ADA 2010 criteria. There were 1732 and 1731 participants included in analysis (A) and analysis (B) respectively. ORs (95% CIs) were adjusted for age, sex, educational attainment (high school or further education, less than high school), current smoking (yes, no), current drinking (yes, no), physical activity (low, moderate, or high), family history of diabetes (yes, no), dyslipidemia (yes, no), hypertension (yes, no), elevated liver enzymes (yes, no), and hyperuricemia (yes, no). ADA, American Diabetes Association; BMI, body mass index; CI, confidence interval; FPG, fasting plasma glucose; HbA1c, glycated hemoglobin; NAFLD, nonalcoholic fatty liver disease; NGT, normal glucose tolerance; NO_[BMI]_ indicates non‐obesity, defined as BMI <28 kg/m^2^; NO_[WC]_ indicates non‐obesity, defined as waist circumference <90 cm for men and <85 cm for women; OB_[BMI]_ indicates general obesity, defined as BMI ≥28 kg/m^2^; OB_[WC]_ indicates abdominal obesity, defined as waist circumference of ≥90 cm in men and ≥85 cm in women; OR, odds ratio; 2‐h PG, 2‐h plasma glucose.

During a median of 4.3 years follow‐up, 452 (26.1%) participants had NAFLD resolved. Prediabetes (multivariable‐adjusted OR 0.66; 95% CI 0.49–0.88), general obesity (0.66; 0.52–0.83), and abdominal obesity (0.73; 0.58–0.91) were independently associated with a decreased risk of resolution of NAFLD (Supplementary Table S5). Regarding diabesity defined by general obesity, compared with the category of NGT with nonobesity, the categories of prediabetes with obesity (ORs ranged from 0.33 to 0.49) and diabetes with obesity (ORs ranged from 0.55 to 0.64) were inversely associated with resolution of NAFLD across various definitions of glucose tolerance status (Figure 3A), with the definition by fasting and 2‐h glucose presenting the lowest ORs. For glucose tolerance status defined by fasting glucose and 2‐h glucose or by fasting glucose and HbA1c, compared with the category of NGT with nonobesity, the categories of prediabetes with nonobesity and NGT with obesity were also associated with decreased risks of resolution of NAFLD.

Similarly, as for diabesity defined by abdominal obesity, compared with the diabesity category of NGT with nonobesity, the category of prediabetes with abdominal obesity presented the lowest OR for the resolution of NAFLD, ranging from 0.42 (0.28–0.62) to 0.57 (0.39–0.84) across different definitions of glucose tolerance status (Figure 3B).

In sensitivity analyses, we observed similar association patterns of diabesity with the resolution of NAFLD among participants aged <75 years (Supplementary Tables S6 and S7).

## 4 DISCUSSION

In this prospective cohort study, we comprehensively delineated the association patterns of diabesity phenotypes with the incidence and resolution of NAFLD. The risk of incident NAFLD associated with diabesity was primarily driven by obesity. Compared with the diabesity category of NGT with nonobesity, the categories of either diabetes or obesity were associated with increased risks of incident NAFLD, and the categories of obesity regardless of glucose tolerance status showed relatively higher risks than the categories of nonobesity. By contrast, the diabesity association pattern with the resolution of NAFLD was mainly driven by the combined phenotype of glucose intolerance and obesity. Compared with the diabesity category of NGT with nonobesity, the categories of prediabetes or diabetes with obesity were associated with decreased likelihoods of a resolution of NAFLD. These association patterns were consistent across various definitions of diabesity based on glucose tolerance status diagnosed by different combinations of glycemic parameters and general or abdominal obesity, supporting the robustness of the main findings.

One main finding of this study was that obesity, particularly general obesity, was the main factor determining the effect of diabesity on incident NAFLD. This observation was in line with a recent Mendelian randomization study that genetically determined BMI (OR per 1‐SD increase of BMI 2.3; 95% CI 2.0–2.7) had a greater effect than type 2 diabetes (OR 1.1; 95% CI 1.0–1.2) on NAFLD.^21^ Potential mechanisms could partially explain our results. Obesity has a central role in contributing to the pathogenesis of NAFLD, not only directly through inflammation, dysregulated release of adipokines, and increased release of free fatty acids that led to intrahepatic fat accumulation,^22^, ^23^ but also indirectly through the same mechanisms that induce diabetes‐associated hyperglycemia and insulin resistance.^6^, ^24^

In addition, although obesity has been documented to be an important risk factor for incident NAFLD,^25^ current evidence of whether general obesity or abdominal obesity is more strongly associated with NAFLD is controversial.^25^, ^26^, ^27^, ^28^ Previous studies suggested that waist circumference is a better predictor of severe liver diseases than BMI because waist circumference has a better ability to identify individuals with increased visceral adipose tissue, which is thought to be biologically more directly linked to metabolic health.^26^, ^27^ On the contrary, two meta‐analyses of observational studies showed that general obesity was associated with a greater NAFLD risk than abdominal obesity.^25^, ^28^ Distinct from abdominal obesity, which signifies visceral fat accumulation, general obesity represents overall adipose tissue deposition throughout the body.^29^ General obesity involves not only the accumulation of visceral fat but also subcutaneous and ectopic fat deposits in various organs, contributing to systemic insulin resistance and lipid metabolism disturbances, which are key pathophysiological mechanisms underlying NAFLD.^30^ The capacity of subcutaneous adipose tissue to store excess energy is diminished in individuals with obesity; consequently, fat deposits outside of subcutaneous tissue, such as into visceral tissue and the liver, and the ectopic fats induce insulin resistance and local inflammation and subsequently contribute to NAFLD.^22^, ^31^ Our study provided new evidence that diabesity defined by general obesity was associated with a more pronounced risk of incident NAFLD than diabesity defined by abdominal obesity, and the consistent results in adults aged <75 years further imply that the main findings of this study might not be biased by possible mass loss in older individuals. Together with previous evidence, our findings suggested that the routine determination of both BMI and waist circumference would be beneficial to comprehensively capture NAFLD risks associated with increased adiposity, highlighting that in the scenario of diabesity, interventions targeting obesity, especially general obesity, are imperative for the primary prevention of NAFLD.

Another noteworthy finding of this study was that NAFLD patients within the diabesity category of prediabetes or diabetes with obesity were less likely to achieve the resolution of NAFLD, especially those with both prediabetes and obesity. Prediabetes is prevalent in NAFLD patients.^32^ In a longitudinal cohort study involving 4273 Chinese adults, we reported that increased fasting and 2‐h glucose levels within the nondiabetic range were inversely associated with the resolution of NAFLD by ultrasound.^20^ However, unlike diabetes, which is a well‐established contributor to the pathophysiology of NAFLD, little attention has been paid to the relationship between prediabetes and the resolution of NAFLD. The association between prediabetes and NAFLD cannot be fully explained by shared risk factors such as obesity and may involve the pathophysiology of hyperglycemia and insulin resistance.^33^ Insulin resistance leads to increased circulating glucose and lipid substrate availability for hepatic lipid accumulation.^33^ Hyperglycemia per se can directly contribute to hepatic steatosis and lipotoxicity by increasing hepatic fat synthesis and inhibiting fat oxidation by activating the hepatic transcription factor carbohydrate response element‐binding protein.^34^ Interestingly, our study revealed that individuals with both prediabetes and obesity had a lower likelihood of achieving a resolution of NAFLD compared to individuals with both diabetes and obesity. This observation might be attributed to the differences in medical management, treatment adherence, and lifestyle modifications between individuals with diabetes and those with prediabetes. Further research is needed to elucidate the precise mechanisms underlying this observation. Our findings underscored the necessity to advance the NAFLD control threshold to prediabetes and proactively manage NAFLD patients with prediabetes or diabetes and obesity.

Strengths of this study included the prospective study design and the comprehensive evaluation of diabesity phenotypes encompassing an extensive profile of glucose tolerance status and obesity, which took individual heterogeneity into account and could facilitate clinical applications for assessing the incidence and resolution of NAFLD in individuals with various glycemic or obesity characteristics. Several limitations also existed in this study. First, the study was conducted on middle‐aged and elderly Chinese adults, and the definition of obesity was appropriate for Asians. Our study facilitates the comparisons of findings with those from other racial groups, but the generalization of our findings to other populations should be cautious. Second, the fatty liver diagnosis was determined using ultrasound rather than histological findings, which might be potentially biased. Nevertheless, ultrasound is the most commonly used method to diagnose fatty liver in clinical practice or large‐scale onsite epidemiological investigations due to its wide availability and affordability.^35^ Third, the relatively short follow‐up duration and only one follow‐up visit limited the ability to evaluate the relationships of the longitudinal trajectories of fasting plasma glucose and BMI with the incidence and resolution of NAFLD. However, we considered age‐related weight loss in the elderly population, and the sensitivity analyses performed among adults aged <75 years further validated the robustness of our findings. Finally, although we have carefully controlled for multiple potential confounders in the analyses and the main findings were highly consistent across various definitions of glucose tolerance status, bias from residual or unmeasured confounders could not be completely eliminated.

In conclusion, this prospective cohort study elaborated that the diabesity association pattern with the incidence of NAFLD was mainly determined by obesity, whereas that with the resolution of NAFLD was primarily driven by the combined phenotype of glucose intolerance and obesity. Our findings shed light on the pathophysiology of NAFLD and suggested diabesity be incorporated into NAFLD management programs to prioritize risk stratifications and guide personalized strategies for the prevention and control of NAFLD.

## FUNDING INFORMATION

This work was supported by the grants from the National Natural Science Foundation of China (82022011, 81970706, 82088102, 81970728), the “Shanghai Municipal Education Commission–Gaofeng Clinical Medicine Grant Support” from Shanghai Jiao Tong University School of Medicine (20171901 Round 2), the Shanghai Municipal Government Grant (22Y31900300), and the Innovative Research Team of High‐level Local Universities in Shanghai.

## CONFLICT OF INTEREST STATEMENT

The authors declared no conflict of interest.

## Supporting information


**Data S1:** Supporting Information.

## Data Availability

Some or all data sets generated during and/or analyzed during the current study are not publicly available but are available from the corresponding author on reasonable request.
