# Supplementary material for: Diabesity phenotype in relation to the incidence and resolution of nonalcoholic fatty liver disease: A prospective cohort study
Source: J Diabetes. 2023 Aug 16;16(1):e13459. doi: 10.1111/1753-0407.13459 (PMC10809295; doi:10.1111/1753-0407.13459)
Supplement: Supplementary file 1 — Data S1: Supporting Information. [file JDB-16-e13459-s001.pdf]

Supplementary files

Table of contents

**Supplementary Table 1.** Baseline characteristics of participants according to diabesity category stratified by baseline NAFLD status	… … … … … 2
**Supplementary Table 2.** ORs (95% CIs) for incident NAFLD associated with glucose intolerance status, general obesity, and abdominal obesity	……………..…5
**Supplementary Table 3.** ORs (95% CIs) for incident NAFLD associated with diabesity category of glucose tolerance status defined by different combinations of glycemic parameters and general obesity status among participants aged <75 years	……………..……6
**Supplementary Table 4.** ORs (95% CIs) for incident NAFLD associated with diabesity category of glucose tolerance status defined by different combinations of glycemic parameters and abdominal obesity status among participants aged <75 years	……………..……8
**Supplementary Table 5.** ORs (95% CIs) for the resolution of NAFLD associated with glucose intolerance status, general obesity, and abdominal obesity	……………10
**Supplementary Table 6.** ORs (95% CIs) for the resolution of NAFLD associated with diabesity category of glucose tolerance status defined by different combinations of glycemic parameters and general obesity status among participants aged <75 years	……………11
**Supplementary Table 7.** ORs (95% CIs) for the resolution of NAFLD associated with diabesity category of glucose tolerance status defined by different combinations of glycemic parameters and abdominal obesity status among participants aged <75 years	… … … … ….13

Supplementary Table 1. Baseline characteristics of participants according to diabesity category[a] stratified by baseline NAFLD status

Characteristic	Normal glucose tolerance		Prediabetes		Diabetes	
	Non-obesity	Abdominal obesity	Non-obesity	Abdominal obesity	Non-obesity	Abdominal obesity
Non-NAFLD (n=3816)[b]						
Participants, n (%)	1410 (37.0)	262 (6.9)	1354 (35.5)	333 (8.7)	343 (9.0)	114 (3.0)
Age, years	54.4 (8.6)	57.1 (8.6)	57.9 (8.7)	59.9 (8.3)	60.3 (8.6)	63.2 (8.7)
Men, n (%)	414 (29.4)	75 (28.6)	387 (28.6)	86 (25.8)	139 (40.5)	40 (35.1)
High school or further education, n (%)	344 (24.4)	45 (17.2)	246 (18.2)	42 (12.6)	81 (23.6)	17 (14.9)
Current cigarette smoker, n (%)	234 (16.6)	30 (11.5)	199 (14.7)	34 (10.2)	55 (16.0)	12 (10.5)
Current alcohol drinker, n (%)	34 (2.4)	8 (3.1)	40 (3.0)	13 (3.9)	12 (3.5)	3 (2.6)
Physical activity, n (%)						
Low	880 (62.4)	175 (66.8)	877 (64.8)	250 (75.1)	226 (65.9)	90 (79.0)
Moderate	297 (21.1)	59 (22.5)	303 (22.4)	59 (17.7)	81 (23.6)	16 (14.0)
High	233 (16.5)	28 (10.7)	174 (12.9)	24 (7.2)	36 (10.5)	8 (7.0)
Family history of diabetes, n (%)	111 (7.9)	19 (7.3)	107 (7.9)	31 (9.3)	81 (23.6)	24 (21.1)
Dyslipidemia, n (%)	385 (27.3)	107 (40.8)	454 (33.5)	142 (42.6)	138 (40.2)	58 (50.9)
Hypertension, n (%)	572 (40.6)	163 (62.2)	735 (54.5)	241 (72.4)	226 (65.9)	99 (86.8)
Elevated liver enzymes, n (%)	55 (3.9)	16 (6.1)	65 (4.8)	27 (8.1)	28 (8.2)	16 (14.0)
Hyperuricemia, n (%)	95 (6.7)	34 (13.0)	124 (9.2)	48 (14.4)	32 (9.3)	19 (16.7)
Medical history, n (%)						
Antihypertensive medications	206 (14.6)	76 (29.0)	286 (21.1)	129 (38.7)	114 (33.2)	50 (43.9)
Glucose-lowering medications	0 (0)	0 (0)	0 (0)	0 (0)	146 (42.6)	62 (54.4)
BMI, kg/m^2	23.3 (2.3)	27.4 (2.1)	23.6 (2.3)	27.6 (2.1)	23.7 (2.4)	27.7 (2.4)
Waist circumference, cm	76.4 (6.1)	89.7 (4.1)	77.3 (6.0)	90.2 (4.1)	79.1 (5.5)	91.4 (4.8)

Fasting plasma glucose, mmol/L	4.8 (0.4)	4.8 (0.4)	5.3 (0.6)	5.2 (0.6)	7.3 (2.3)	7.1 (1.9)
OGTT 2-h plasma glucose, mmol/L	5.7 (1.1)	5.9 (1.1)	7.1 (1.8)	7.5 (1.7)	14.5 (5.7)	14.7 (4.6)
HbA1c, %	5.3 (0.2)	5.4 (0.2)	5.7 (0.3)	5.8 (0.3)	6.8 (1.5)	6.9 (1.3)
HOMA-IR	1.2 (0.8-1.6)	1.6 (1.2-2.1)	1.3 (0.9-1.8)	1.8 (1.3-2.5)	1.8 (1.2-2.8)	2.8 (1.9-4.0)
NAFLD (n=1731)[c]						
Participants, n (%)	136 (7.9)	194 (11.2)	318 (18.4)	461 (26.6)	223 (12.9)	399 (23.1)
Age, years	53.3 (6.6)	55.7 (8.2)	56.9 (7.3)	57.9 (8.4)	58.0 (8.2)	59.6 (8.4)
Men, n (%)	47 (34.6)	68 (35.1)	92 (28.9)	121 (26.3)	75 (33.6)	136 (34.1)
High school or further education, n (%)	41 (30.2)	46 (23.7)	58 (18.2)	87 (18.9)	48 (21.5)	67 (16.8)
Current cigarette smoker, n (%)	28 (20.6)	29 (15.0)	45 (14.2)	60 (13.0)	34 (15.3)	76 (19.1)
Current alcohol drinker, n (%)	4 (2.9)	8 (4.1)	13 (4.1)	12 (2.6)	6 (2.7)	19 (4.8)
Physical activity, n (%)						
Low	82 (60.3)	125 (64.4)	219 (68.9)	314 (68.1)	151 (67.7)	285 (71.4)
Moderate	29 (21.3)	43 (22.2)	63 (19.8)	87 (18.9)	42 (18.8)	79 (19.8)
High	25 (18.4)	26 (13.4)	36 (11.3)	60 (13.0)	30 (13.5)	35 (8.8)
Family history of diabetes, n (%)	17 (12.5)	21 (10.8)	43 (13.5)	48 (10.4)	43 (19.3)	94 (23.6)
Dyslipidemia, n (%)	77 (56.6)	116 (59.8)	201 (63.2)	268 (58.1)	146 (65.5)	254 (63.7)
Hypertension, n (%)	74 (54.4)	129 (66.5)	213 (67.0)	365 (79.2)	177 (79.4)	322 (80.9)
Elevated liver enzymes, n (%)	14 (10.3)	26 (13.4)	37 (11.6)	81 (17.6)	37 (16.6)	119 (29.8)
Hyperuricemia, n (%)	19 (14.0)	55 (28.4)	56 (17.6)	123 (26.7)	51 (22.9)	107 (26.8)
Medical history, n (%)						
Antihypertensive medications	29 (21.3)	65 (33.5)	105 (33.0)	205 (44.5)	84 (37.7)	200 (50.1)
Glucose-lowering medications	0 (0)	0 (0)	0 (0)	0 (0)	76 (34.1)	127 (31.8)
BMI, kg/m^2	25.5 (2.1)	28.6 (2.5)	25.4 (1.9)	28.9 (2.6)	25.2 (2.0)	29.1 (3.1)
Waist circumference, cm	82 (4.0)	92.5 (5.2)	81.2 (4.3)	92.6 (5.5)	82.1 (4.0)	94.3 (6.8)

Fasting plasma glucose, mmol/L	4.9 (0.4)	4.9 (0.4)	5.4 (0.6)	5.4 (0.6)	7.7 (2.6)	7.6 (2.6)
OGTT 2-h plasma glucose, mmol/L	6 (1.2)	6.2 (1.0)	7.8 (1.6)	7.9 (1.7)	15.9 (5.7)	15.2 (4.9)
HbA1c, %	5.3 (0.2)	5.4 (0.2)	5.8 (0.3)	5.8 (0.3)	7.2 (1.5)	7.2 (1.5)
HOMA-IR	1.7 (1.1-2.2)	2.2 (1.7-2.9)	2.0 (1.5-2.8)	2.7 (2.0-3.6)	2.9 (1.9-4.4)	4.0 (2.6-5.7)

[a]Diabesity categories were based on three glycemic parameters-defined glucose tolerance status according to the ADA criteria 2010 and abdominal obesity.

Data are mean (SD) for continuous variables with normal distribution, median (interquartile range) for continuous variables with skewed distribution, and number (proportion) for categorical variables.

[b]The number of missing data was 3 for 2-h plasma glucose and 4 for HbA1c.

[c]The number of missing data was 2 for 2-h plasma glucose and 2 for HbA1c.

Abbreviations: ADA, American Diabetes Association; BMI, body mass index; HbA1c, glycated hemoglobin; HOMA-IR, homeostasis model assessment of insulin resistance; NAFLD, nonalcoholic fatty liver disease; OGTT, oral glucose tolerance test.

Supplementary Table 2. ORs (95% CIs) for incident NAFLD associated with glucose intolerance status, general obesity, and abdominal obesity

Variable	Participants, n	Cases, n (%)	OR (95% CI)	
			Model 1	Model 2
Glucose tolerance status[a]				
Normal glucose tolerance	1673	259 (15.5)	1.00 (Ref)	1.00 (Ref)
Prediabetes	1687	326 (19.3)	1.31 (1.09-1.57)	1.26 (1.05-1.52)
Diabetes	457	100 (21.9)	1.45 (1.10-1.91)	1.35 (1.03-1.78)
General obesity				
No	3463	543 (15.7)	1.00 (Ref)	1.00 (Ref)
Yes	354	142 (40.1)	3.56 (2.81-4.51)	3.28 (2.58-4.17)
Abdominal obesity				
No	3107	469 (15.1)	1.00 (Ref)	1.00 (Ref)
Yes	709	216 (30.5)	2.42 (1.99-2.93)	2.27 (1.87-2.76)

[a]Glucose tolerance status were defined based on a self-reported previous diagnosis of diabetes by health-care professionals and fasting plasma glucose, OGTT 2-h plasma glucose, and HbA1c, according to the ADA criteria 2010.

Model 1 was adjusted for age, sex, educational attainment (high school or further education, less than high school), current smoking (yes, no), current drinking (yes, no), physical activity (low, moderate, or high), family history of diabetes (yes, no), and dyslipidemia (yes, no).

Model 2 was further adjusted for hypertension (yes, no), elevated liver enzymes (yes, no), and hyperuricemia (yes, no), based on Model 1.

Abbreviations: ADA, American Diabetes Association; CI, confidence interval; HbA1c, glycated hemoglobin; NAFLD, nonalcoholic fatty liver disease; OGTT, oral glucose tolerance test; OR, odds ratio.

Supplementary Table 3. ORs (95% CIs) for incident NAFLD associated with diabesity category of glucose tolerance status defined by different combinations of glycemic parameters and general obesity status among participants aged <75 years

	**Normal glucose tolerance**		**Prediabetes**		**Diabetes**	
**Diabesity category[a]**	**Non-obesity**	**General obesity**	**Non-obesity**	**General obesity**	**Non-obesity**	**General obesity**
Glucose tolerance status defined by FPG, 2-h PG, and HbA1c						
Participants, n	1508	125	1450	159	368	56
Cases, n (%)	208 (13.8)	45 (36.0)	245 (16.9)	71 (44.7)	74 (20.1)	21 (37.5)
ORs (95% CIs)[b]	1.00 (Ref)	3.15 (2.10-4.73)	1.24 (1.01-1.52)	4.63 (3.24-6.62)	1.42 (1.04-1.94)	3.16 (1.76-5.65)
Glucose tolerance status defined by FPG and 2-h PG						
Participants, n	2140	200	840	87	346	53
Cases, n (%)	308 (14.4)	78 (39.0)	148 (17.6)	41 (47.1)	71 (20.5)	18 (34.0)
ORs (95% CIs)[b]	1.00 (Ref)	3.45 (2.51-4.74)	1.19 (0.96-1.49)	4.76 (3.03-7.49)	1.38 (1.01-1.87)	2.56 (1.40-4.66)
Glucose tolerance status defined by FPG and HbA1c						
Participants, n	1744	144	1292	147	287	49
Cases, n (%)	250 (14.3)	52 (36.1)	218 (16.9)	67 (45.6)	59 (20.6)	18 (36.7)
ORs (95% CIs)[b]	1.00 (Ref)	3.07 (2.10-4.47)	1.18 (0.96-1.45)	4.51 (3.14-6.49)	1.37 (0.98-1.92)	2.91 (1.57-5.38)
Glucose tolerance status defined by 2-h PG and HbA1c						
Participants, n	1641	131	1332	155	353	54
Cases, n (%)	224 (13.7)	46 (35.1)	235 (17.6)	70 (45.2)	68 (19.3)	21 (38.9)
ORs (95% CIs)[b]	1.00 (Ref)	3.11 (2.09-4.63)	1.34 (1.09-1.64)	4.77 (3.34-6.83)	1.36 (0.99-1.87)	3.45 (1.92-6.21)

There were 3666 participants included in the analysis. The number of missing data was 3 for the analysis of definition by FPG and HbA1c.

[a]For all diabesity categories, glucose tolerance status was defined by different combinations of glycemic parameters along with a self-reported previous diagnosis of diabetes by health care professionals, according to the ADA 2010 criteria.

[b]OR (95% CIs) were adjusted for age, sex, educational attainment (high school or further education, less than high school), current smoking (yes, no), current drinking (yes, no), physical activity (low, moderate, or high), family history of diabetes (yes, no), dyslipidemia (yes, no), hypertension (yes, no), elevated liver enzymes (yes, no), and hyperuricemia (yes, no).

Abbreviations: ADA, American Diabetes Association; CI, confidence interval; FPG, fasting plasma glucose; HbA1c, glycated hemoglobin; NAFLD, nonalcoholic fatty liver disease; OR, odds ratio; 2-h PG, 2-h plasma glucose.

Supplementary Table 4. ORs (95% CIs) for incident NAFLD associated with diabesity category of glucose tolerance status defined by different combinations of glycemic parameters and abdominal obesity status among participants aged <75 years

Diabesity category[a]	Normal glucose tolerance		Prediabetes		Diabetes	
	Non-obesity	Abdominal obesity	Non-obesity	Abdominal obesity	Non-obesity	Abdominal obesity
Glucose tolerance status defined by FPG, 2-h PG, and HbA1c						
Participants, n	1378	254	1292	317	323	101
Cases, n (%)	186 (13.5)	67 (26.4)	208 (16.1)	108 (34.1)	65 (20.1)	30 (29.7)
ORs (95% CIs)[b]	1.00 (Ref)	2.09 (1.51-2.90)	1.21 (0.97-1.50)	3.07 (2.29-4.12)	1.49 (1.07-2.07)	2.29 (1.42-3.69)
Glucose tolerance status defined by FPG and 2-h PG						
Participants, n	1949	390	738	189	306	93
Cases, n (%)	276 (14.2)	110 (28.2)	120 (16.3)	69 (36.5)	63 (20.6)	26 (28)
ORs (95% CIs)[b]	1.00 (Ref)	2.19 (1.69-2.85)	1.11 (0.88-1.41)	3.11 (2.21-4.36)	1.44 (1.04-1.98)	1.99 (1.22-3.25)
Glucose tolerance status defined by FPG and HbA1c						
Participants, n	1583	304	1155	284	252	84
Cases, n (%)	223 (14.1)	79 (26)	183 (15.8)	102 (35.9)	53 (21)	24 (28.6)
ORs (95% CIs)[b]	1.00 (Ref)	1.97 (1.46-2.67)	1.13 (0.91-1.40)	3.10 (2.31-4.16)	1.48 (1.04-2.10)	2.06 (1.23-3.45)
Glucose tolerance status defined by 2-h PG and HbA1c						
Participants, n	1506	265	1180	307	307	100
Cases, n (%)	200 (13.3)	70 (26.4)	199 (16.9)	106 (34.5)	60 (19.5)	29 (29)
ORs (95% CIs)[b]	1.00 (Ref)	2.16 (1.57-2.97)	1.32 (1.06-1.64)	3.21 (2.40-4.30)	1.47 (1.05-2.06)	2.28 (1.41-3.68)

There were 3665 participants included in the analysis. The number of missing data was 3 for the analysis of definition by FPG and HbA1c.

[a]For all diabesity categories, glucose tolerance status was defined by different combinations of glycemic parameters along with a self-reported previous diagnosis of diabetes by health care professionals, according to the ADA 2010 criteria.

[b]OR (95% CIs) were adjusted for age, sex, educational attainment (high school or further education, less than high school), current smoking (yes, no), current drinking (yes, no), physical activity (low, moderate, or high), family history of diabetes (yes, no), dyslipidemia (yes, no), hypertension (yes, no), elevated liver enzymes (yes, no), and hyperuricemia (yes, no).

Abbreviations: ADA, American Diabetes Association; CI, confidence interval; FPG, fasting plasma glucose; HbA1c, glycated hemoglobin; NAFLD, nonalcoholic fatty liver disease; OR, odds ratio; 2-h PG, 2-h plasma glucose.

Supplementary Table 5. ORs (95% CIs) for the resolution of NAFLD associated with glucose intolerance status, general obesity, and abdominal obesity

Variable	Participants, n	Cases, n (%)	OR (95% CI)	
			Model 1	Model 2
Glucose tolerance status[a]				
Normal glucose tolerance	330	104 (31.5)	1.00 (Ref)	1.00 (Ref)
Prediabetes	779	182 (23.4)	0.64 (0.48-0.86)	0.66 (0.49-0.88)
Diabetes	623	166 (26.7)	0.74 (0.55-1.00)	0.79 (0.58-1.07)
General obesity				
No	1029	305 (29.6)	1.00 (Ref)	1.00 (Ref)
Yes	703	147 (20.9)	0.63 (0.50-0.78)	0.66 (0.52-0.83)
Abdominal obesity				
No	677	204 (30.1)	1.00 (Ref)	1.00 (Ref)
Yes	1054	248 (23.5)	0.69 (0.56-0.86)	0.73 (0.58-0.91)

[a]Glucose tolerance status were defined based on a self-reported previous diagnosis of diabetes by health-care professionals and fasting plasma glucose, OGTT 2-h plasma glucose, and HbA1c, according to the ADA criteria 2010.

Model 1 was adjusted for age, sex, educational attainment (high school or further education, less than high school), current smoking (yes, no), current drinking (yes, no), physical activity (low, moderate, or high), family history of diabetes (yes, no), and dyslipidemia (yes, no).

Model 2 was further adjusted for hypertension (yes, no), elevated liver enzymes (yes, no), and hyperuricemia (yes, no), based on Model 1.

Abbreviations: ADA, American Diabetes Association; CI, confidence interval; HbA1c, glycated hemoglobin; NAFLD, nonalcoholic fatty liver disease; OGTT, oral glucose tolerance test; OR, odds ratio.

Supplementary Table 6. ORs (95% CIs) for the resolution of NAFLD associated with diabesity category of glucose tolerance status defined by different combinations of glycemic parameters and general obesity status among participants aged <75 years

	**Normal glucose tolerance**		**Prediabetes**		**Diabetes**	
**Diabesity category[a]**	**Non-obesity**	**General obesity**	**Non-obesity**	**General obesity**	**Non-obesity**	**General obesity**
Glucose tolerance status defined by FPG, 2-h PG, and HbA1c						
Participants, n	197	129	453	302	348	248
Cases, n (%)	66 (33.5)	36 (27.9)	127 (28)	50 (16.6)	101 (29)	56 (22.6)
ORs (95% CIs)[b]	1.00 (Ref)	0.79 (0.48-1.29)	0.76 (0.53-1.10)	0.41 (0.26-0.63)	0.79 (0.54-1.17)	0.59 (0.38-0.93)
Glucose tolerance status defined by FPG and 2-h PG						
Participants, n	322	214	347	232	329	233
Cases, n (%)	114 (35.4)	55 (25.7)	83 (23.9)	34 (14.7)	97 (29.5)	53 (22.8)
ORs (95% CIs)[b]	1.00 (Ref)	0.65 (0.44-0.95)	0.57 (0.41-0.81)	0.33 (0.21-0.51)	0.75 (0.54-1.06)	0.55 (0.37-0.83)
Glucose tolerance status defined by FPG and HbA1c						
Participants, n	289	192	435	295	274	192
Cases, n (%)	96 (33.2)	41 (21.4)	115 (26.4)	57 (19.3)	83 (30.3)	44 (22.9)
ORs (95% CIs)[b]	1.00 (Ref)	0.56 (0.36-0.86)	0.70 (0.50-0.98)	0.49 (0.33-0.73)	0.85 (0.59-1.23)	0.62 (0.40-0.95)
Glucose tolerance status defined by 2-h PG and HbA1c						
Participants, n	223	150	436	288	339	241
Cases, n (%)	73 (32.7)	38 (25.3)	121 (27.8)	48 (16.7)	100 (29.5)	56 (23.2)
ORs (95% CIs)[b]	1.00 (Ref)	0.72 (0.45-1.15)	0.78 (0.55-1.12)	0.43 (0.28-0.66)	0.85 (0.58-1.24)	0.65 (0.42-1.00)

There were 1677 participants included in the analysis.

[a]For all diabesity categories, glucose tolerance status was defined by different combinations of glycemic parameters along with a self-reported previous diagnosis of diabetes by health care professionals, according to the ADA 2010 criteria.

[b]OR (95% CIs) were adjusted for age, sex, educational attainment (high school or further education, less than high school), current smoking (yes, no), current drinking (yes, no), physical activity (low, moderate, or high), family history of diabetes (yes, no), dyslipidemia (yes, no),

hypertension (yes, no), elevated liver enzymes (yes, no), and hyperuricemia (yes, no).

Abbreviations: ADA, American Diabetes Association; CI, confidence interval; FPG, fasting plasma glucose; HbA1c, glycated hemoglobin; NAFLD, nonalcoholic fatty liver disease; OR, odds ratio; 2-h PG, 2-h plasma glucose.

Supplementary Table 7. ORs (95% CIs) for the resolution of NAFLD associated with diabesity category of glucose tolerance status defined by different combinations of glycemic parameters and abdominal obesity status among participants aged <75 years

Diabesity category[a]	Normal glucose tolerance		Prediabetes		Diabetes	
	**Non-obesity**	**Abdominal obesity**	**Non-obesity**	**Abdominal obesity**	**Non-obesity**	**Abdominal obesity**
Glucose tolerance status defined by FPG, 2-h PG, and HbA1c						
Participants, n	136	190	315	440	216	379
Cases, n (%)	43 (31.6)	59 (31.1)	90 (28.6)	87 (19.8)	69 (31.9)	88 (23.2)
ORs (95% CIs)[b]	1.00 (Ref)	1.00 (0.62-1.61)	0.84 (0.54-1.32)	0.55 (0.35-0.85)	1.03 (0.64-1.65)	0.65 (0.41-1.02)
Glucose tolerance status defined by FPG and 2-h PG						
Participants, n	218	318	247	332	202	359
Cases, n (%)	76 (34.9)	93 (29.3)	59 (23.9)	58 (17.5)	67 (33.2)	83 (23.1)
ORs (95% CIs)[b]	1.00 (Ref)	0.78 (0.54-1.14)	0.58 (0.38-0.87)	0.41 (0.28-0.62)	0.94 (0.62-1.42)	0.56 (0.38-0.82)
Glucose tolerance status defined by FPG and HbA1c						
Participants, n	195	286	300	430	172	293
Cases, n (%)	62 (31.8)	75 (26.2)	84 (28)	88 (20.5)	56 (32.6)	71 (24.2)
ORs (95% CIs)[b]	1.00 (Ref)	0.78 (0.52-1.17)	0.81 (0.55-1.21)	0.56 (0.38-0.83)	1.03 (0.66-1.62)	0.69 (0.45-1.05)
Glucose tolerance status defined by 2-h PG and HbA1c						
Participants, n	156	217	300	424	211	368
Cases, n (%)	49 (31.4)	62 (28.6)	85 (28.3)	84 (19.8)	68 (32.2)	88 (23.9)
ORs (95% CIs)[b]	1.00 (Ref)	0.90 (0.57-1.41)	0.85 (0.55-1.30)	0.56 (0.37-0.85)	1.06 (0.67-1.67)	0.69 (0.44-1.06)

There were 1676 participants included in the analysis.

[a]For all diabesity categories, glucose tolerance status was defined by different combinations of glycemic parameters along with a self-reported previous diagnosis of diabetes by health care professionals, according to the ADA 2010 criteria.

[b]OR (95% CIs) were adjusted for age, sex, educational attainment (high school or further education, less than high school), current smoking (yes, no), current drinking (yes, no), physical activity (low, moderate, or high), family history of diabetes (yes, no), dyslipidemia (yes, no), hypertension (yes, no), elevated liver enzymes (yes, no), and hyperuricemia (yes, no).

Abbreviations: ADA, American Diabetes Association; CI, confidence interval; FPG, fasting plasma glucose; HbA1c, glycated hemoglobin; NAFLD, nonalcoholic fatty liver disease; OR, odds ratio; 2-h PG, 2-h plasma glucose.
